# Development and Validation of a New DIVA Real-Time PCR Allowing to Differentiate Wild-Type Lumpy Skin Disease Virus Strains, Including the Asian Recombinant Strains, from Neethling-Based Vaccine Strains

**DOI:** 10.3390/v15040870

**Published:** 2023-03-28

**Authors:** Andy Haegeman, Ilse De Leeuw, Wannes Philips, Nick De Regge

**Affiliations:** 1 Sciensano, Infectious Diseases in Animals, Exotic and Vector-Borne Diseases, Groeselenberg 99, B-1180 Brussels, Belgium; 2 Sciensano, EURL for Diseases Caused by Capripox Viruses, Groeselenberg 99, B-1180 Brussels, Belgium

**Keywords:** real-time PCR, lumpy skin disease, DIVA, recombinant strain, vaccine strain

## Abstract

The current epidemic in Asia, driven by LSDV recombinants, poses difficulties to existing DIVA PCR tests, as these do not differentiate between homologous vaccine strains and the recombinant strains. We, therefore, developed and validated a new duplex real-time PCR capable of differentiating Neethling-based vaccine strains from classical and recombinant wild-type strains that are currently circulating in Asia. The DIVA potential of this new assay, seen in the in silico evaluation, was confirmed on samples from LSDV infected and vaccinated animals and on isolates of LSDV recombinants (n = 12), vaccine (n = 5), and classic wild-type strains (n = 6). No cross-reactivity or a-specificity with other capripox viruses was observed under field conditions in non-capripox viral stocks and negative animals. The high analytical sensitivity is translated into a high diagnostic specificity as more than 70 samples were all correctly detected with Ct values very similar to those of a published first-line pan capripox real-time PCR. Finally, the low inter- and intra-run variability observed shows that the new DIVA PCR is very robust which facilitates its implementation in the lab. All validation parameters that are mentioned above indicate the potential of the newly developed test as a promising diagnostic tool which could help to control the current LSDV epidemic in Asia.

## 1. Introduction

Lumpy skin disease virus (LSDV) is the causative agent of lumpy skin disease (LSD), a WOAH notifiable disease with significant socio-economic impacts on the local and international level [1,2]. The virus belongs to the genus of capripox of the family Poxviridae and has a double stranded genome of approximately 150 kbp [3]. Since the first documented cases in Zambia (1925), the disease remained confined for a long time to southern Africa, where it is still endemic to date, although a northward spread can be noted. After reaching the Mediterranean Basin in 1988 [4], it continued spreading towards the Middle East with outbreaks in 2013 in Turkey [5]. Europe followed suit with large-scale outbreaks in the Balkan region in 2015/16 [6]. Simultaneously, there was an eastward spread of the virus towards the northern Caucasus region and the virus entered the Russian Federation in 2016 [7]. In 2019, its introduction was reported in Bangladesh [8], China [9,10], and India [11]. Since its introduction in Asia, multiple countries have reported LSDV outbreaks from 2020 onwards, such as Nepal [12], Vietnam [13], Mongolia [14], Thailand [15], and Myanmar [16].

Up until 2017, sequence analyses of different LSDV field isolates, separated over time and geographical location, revealed only limited genomic variation [17,18,19]. This suggested that the LSDV genome is relatively stable with two phylogenic clusters, namely the Neethling-based (vaccines) strains (cluster 1.1) and the wild-type strains (hereafter referred to as classical wild-type strains; cluster 1.2) [20]. This changed in 2018 with a report describing the genomic analysis of a vaccine-like isolate that was obtained from Saratovskaya oblast in 2017 from a cow displaying typical LSDV symptoms. This isolate is now referred to as the Saratov 2017 isolate [21]. In this study, multiple recombination sites were found and one of the parental strains was demonstrated to be the Neethling vaccine strain used in the homologous live attenuated vaccines. Following the initial discovery of an LSDV recombinant, new sequencing efforts have shown the existence of multiple recombinant clusters, tentatively named 2.1 to 2.5 [22,23]. Although the exact origin of these recombinant LSDV strains is difficult to prove, there is a tentative link with an LSDV vaccine that is used in that region. When this vaccine was submitted to a quality control evaluation [24], multiple different capripox viruses, including field and vaccine-type LSDV, and recombinants were found to be present. This was confirmed by full genome sequencing of the capripox viruses that were present in the vaccine. This analysis showed not only the presence of a Neethling vaccine strain, a KSGP-based vaccine strain, and even a Sudan-like goatpox strain but importantly, various other different KSGP/Neethling recombinants. The fact that the latter are almost identical to those found in the field leads to the hypothesis that the current recombinant LSDV strain spreading in Asia is likely caused by vaccine spillover [25].

Vaccination has been a useful tool in fighting any disease and this holds also true for LSDV. Live attenuated homologous vaccines have been used for many years in South-Africa against LSDV and were more recently successfully applied in the Balkan region [26]. Their efficacy in the field was confirmed under controlled conditions in BLS3 animal facilities [27]. However, the sustainable application of vaccination is determined by a number of factors of which safety and efficacy are most often placed in the spotlight. The importance of accompanying diagnostics, on the other hand, is often overlooked. The ability to distinguish infected from vaccinated animals, often referred to as the DIVA principle, is crucial in any control/eradication strategy and from an economical (trade) point of view. Due to its sensitivity, specificity, and rapidity, (real-time) PCR-based technologies are at the forefront of diagnostics. The development of DIVA PCR-based tests for LSDV has been facilitated by the fact that the most commonly used commercial live attenuated homologous vaccines are all based on the Neethling vaccine strain. Therefore, it is not surprising that there are a number of DIVA real-time PCRs available, either commercially (for example: ID Gene™ LSD DIVA Triplex kit and Bio-T kit^®^ LSD—DIVA) or published in the public domain [28,29].

The emergence of the recombinant strains, however, has important implications for LSDV diagnostics. This was demonstrated by a vaccination/challenge experiment with the vaccine containing the recombinant strains. PCR analysis of blood and biopsies collected during the post-vaccination period resulted in ambiguous or contradictory conclusions of the LSDV status (vaccinated or infected) depending on the DIVA PCR that was used [24]. A comparative study, including commercially available or published DIVA PCR assays, clearly highlighted the problem further as these tests either did not detect the recombinants, misidentified them as vaccine, or detected them as both vaccine and wild-type LSDV strains [30]. The inability of existing DIVA PCRs to differentiate animals that were vaccinated with Neethling-based vaccines from the circulating virulent recombinant strains poses a great problem in combating the current LSDV epidemic in Asia.

It was, therefore, the purpose of this study to develop and validate a new DIVA real-time PCR able to differentiate the Neethling vaccine strain from the LSDV recombinant strains currently circulating in Asia. This new DIVA real-time PCR is to be used as a typing assay in combination with a first-line general capripox (or LSDV) PCR assay.

## 2. Materials and Methods

### 2.1. Primer and Probe Design and In Silico Analysis

A total of 42 capripox virus sequences, including 29 LSDV, 7 GTPV, and 6 SPPV sequences were aligned, edited, and visualized using the BioEdit software ([31], version 7.0.5.3). Primers and probes targeting conserved regions in the DNA-ligase-like protein (LD133, wild-type reaction [WTR]), and in a kelch-like (LD144; vaccine-type reaction [VR]) genes were designed using the primer3 software ([32]; source code available at http://fokker.wi.mit.edu/primer3/; accessed on 25 January 2022).

### 2.2. Real-Time PCRs

#### 2.2.1. New Duplex DIVA Real-Time PCR

The new DIVA real-time PCR is a duplex consisting of a wild-type reaction (WTR), detecting the classic LSDV wild-type strains and the recombinant LSDV strains that are currently circulating in Asia, and a Neethling-like vaccine reaction (VR). The real-time PCR is carried out in a total volume of 20 µL, consisting of 10 µL LightCycler 480 Probes Master (Roche), 2.5 µL DNA template, 1 U FastStartTaq, 0.1 µg/µL BSA, 0.8 mM MgCl^2^, 0.62 µM of the four primers, and 0.35 µM of both probes. The primers and DNA template were denatured separately at 95 °C for 3 min before the rest of the mix was added. The LightCycler 480 thermal cycling profile for the real-time PCRs was: 95 °C for 10 min and 50 cycles of 95 °C for 10 s and 60 °C for 30 s. Fluorescence acquisition was performed at the end of each annealing/extension step. The primers and probes were synthesized and standard desalted by Integrated DNA Technologies (Leuven, Belgium).

#### 2.2.2. Additional Real-Time PCRs

A previously published diagnostic pan capripox real-time PCR D5R [33] was used to confirm the capripox virus status of some of the samples. In addition, a DIVA PCR described by Agianniotaki et al. (2017) [28] was used for differentiation between the Neethling LSDV vaccine and classical wild-type LSDV strains.

### 2.3. Viruses and Samples Used in the Evaluation of the DIVA Real-Time PCR

For the study of the linearity, the limit of detection (LOD) and the inter/intra run variability, three LSD viruses were used: a classical wild-type LSDV strain from Bulgaria (titer 10^5.2^ TCID_50_/100 µL), a Neethling-like vaccine strain (titer 10^5^ TCID_50_/100 µL), and a recent recombinant wild-type strain from Vietnam (titer 10^6.2^ TCID_50_/100 µL).

For the diagnostic sensitivity, samples were used from previous animal trials conducted in our BSL3 animal facilities (Ethical permits: 20150605-01 and 20170510-01) using Belgian cattle. All of the animals tested negative for capripox prior to the trial and were, therefore, considered negative. For LSDV wild-type positive samples, 4 blood and 36 organ samples were used, obtained from cattle which were infected with LSDV Israel at the start of the trial. There were three additional samples that were included from vaccinated/infected animals. All of these samples were positive on the capripox diagnostic PCR D5R and the wild-type status of the latter were confirmed by the DIVA Agianniotaki [28]. For the vaccine-type positive samples, 34 organ samples were used from animals which were only inoculated with a Neethling-like vaccine during the trials. The status of the Neethling-positive samples were confirmed with the DIVA Agianniotaki [28].

A total of 50 blood samples and 10 skin samples from LSDV-negative Belgian cattle were collected during a previous animal experiment (ethical permit: 20200302-01) and used to determine the diagnostic specificity. The negative status of these samples was determined with the diagnostic real-time PCR D5R.

Virus stocks of non-capripox viruses, classical wild-type LSDV strains, sheeppox and goatpox viruses, recombinant wild-type strains from Vietnam, and Neethling-based vaccines were used to assess the analytical sensitivity (inclusivity/exclusivity) and specificity. The status of these samples had previously been determined using virus-specific diagnostic real-time PCRs assays. In addition, 12 SPPV-positive ovine blood samples, skin samples, and muscle (musculus masseter) samples were analyzed. These ovine samples were collected during a previous animal trial whereby sheep were infected with either Moroccan, Kenyan, Nigeria, and Turkish SPPV strains (ethical dossier 20200622-01) and were tested positive with the pan capripox diagnostic PCR D5R.

To study the impact of the joint presence of wild-type and vaccine strains on the performance of DIVA real-time PCR, DNA samples of the classical wild-type LSDV strain Bulgaria, a Neethling vaccine strain, and a recombinant wild-type isolate of Vietnam were mixed at different ratios and analyzed.

## 3. Results

### 3.1. Primer/Probe Design and In Silico Evaluation

The DIVA real-time PCR was designed as a duplex PCR to detect Neethling-based vaccine strains on the one hand (hereafter referred to as the vaccine reaction (VR)) and wild-type field LSDV strains, including both classical and recombinant wild-type strains on the other hand (referred to as the wild-type reaction (WTR)). The primers and probes used for the detection of wild-type (WT) strains and Neethling vaccine strains were designed in conserved regions of a DNA-ligase-like (LD133) gene and a kelch-like protein (LD144) gene, respectively. The regions were identified after alignment of 42 capripox genomes, including 29 LSDV (5 Neethling vaccine strains, 11 classic wild-type strains, 13 recombinant strains originating from different Asian countries), 7 GTPV, and 6 SPPV sequences. The selected primers and probes are summarized in Table 1.

The forward primer of the WTR is relatively well conserved within the capripox genus (Figure S1). Only a single nucleotide difference is observed in the middle of the forward primer in most of the new recombinant LSDV strains. However, in view of the location of this mismatch in the primer sequence, its estimated impact on the potential primer attachment is limited. More DIVA discrimination power can be found in the reverse primer. While completely conserved in the classical wild-type and recombinant strains, three of the four nucleotides at the 3′end, including the last nucleotide, are mismatched with the Neethling-based vaccine strains. Goatpox viruses have two mismatches in the 3′ terminal region of the primer (last and fourth last nucleotide) while sheeppox viruses have mismatches in the middle and at the 5′ and 3′ end. The probe of the WTR is relatively well conserved among sheeppox, goatpox, and classical and recombinant wild-type LSDV strains while an important mismatch is present with the vaccine strains in the first base of the 5′end. A second, less destabilizing mismatch, is present in the middle of the probe. The latter can also be found in the recombinant strains that were detected in the Saratov region.

The forward primer, reverse primer, and probe of the VR (Figure S2) are completely homologous with the Neethling vaccine strains. The forward primer has several mismatches with classical and recombinant wild-type LSDV strains and with sheeppox and goatpox virus strains. The 5′end of the probe is equally differentiating as the first two nucleotides at the 5′end are mismatched with all capripox viruses except for the Neethling-like vaccine strains. The reverse primer has only two nucleotide differences with classical and recombinant wild-type LSDV strains and goatpox viruses. Only one mismatch is present for sheeppox and this is in the middle of the primer where the effect on the stability and elongation of the primer is less pronounced.

### 3.2. Linear Range and Limit of Detection

**Figure 1:**
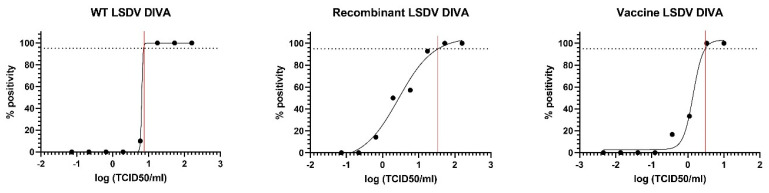
The non-linear regression analysis to determine the limit of detection of the DIVA PCR for classical wild-type, recombinant wild-type, and Neethling vaccine LSDV strains.

The linearity was studied by testing a 10-fold dilution series of a classical wild-type LSDV strain, a recent recombinant wild-type LSDV strain from Asia (Vietnam), and a Neethling vaccine strain. A total of four replicates of each dilution series were tested, resulting in correlation coefficients (R^2^) of 0.9956, 0.9985, and 1, respectively, with the corresponding efficiencies of 109.9%, 100.3 %, and 95.7% (Figure S3). Based on these results, additional three-fold dilution series were generated to determine the limit of detection in more detail, covering a range of concentrations equaling 100% positivity to 100% negativity. A total of ten replicates of each dilution were tested with the new DIVA real-time PCR. Using a nonlinear regression analysis, the LOD was calculated for all three types of LSDV strains and visualized in Figure 1. The obtained LOD for classical, recombinant, and vaccine LSDV strains was 7.3, 34.1, and 3.05 TCID_50_/100 µL, respectively.

### 3.3. Diagnostic and Analytical Parameters

To test the diagnostic sensitivity, 43 blood/organ samples from cattle that were infected with an LSDV classical wild-type strain were analyzed with the DIVA real-time PCR and compared to the diagnostic pan capripox real-time PCR D5R. All 43 samples were correctly characterized as wild-type by the new DIVA. The Ct values of the DIVA PCR were highly comparable, but slightly higher than those of the D5R reaction, with an average difference of 0.89 Ct (standard deviation [SD] 0.84; Table S1). In a similar approach, the organs of Neethling-vaccinated cattle (n = 34) were tested and compared to the D5R. All samples were correctly identified as Neethling-like vaccine by the DIVA, including 2 samples which were negative on the DIVA Agianniotaki. The Ct values of the DIVA PCR were in general somewhat lower compared to those of D5R with an average difference of 1.5 Ct (SD 1.13; Table S2, Figure S4). Fifty blood samples and 10 skin samples of Belgian cattle were used as negatives and analyzed with the DIVA PCR. No false positives were observed as all samples were correctly identified as negative by the new DIVA PCR, resulting in a DSp of 100%.

To check the analytical specificity (ASp), potential cross reactions with 16 non-capripox viruses were analyzed. This panel included five parapox field isolates (collected between 1987 and 2005), three bluetongue viruses (serotype 2, 4, and 8), three foot-and-mouth disease viruses (serotype C, O, and Asia), two vesicular stomatitis viruses (Indiana and New Jersey type), and one schmallenberg virus, one bovine viral diarrhea virus (Type 1), and ovine herpesvirus 1. All these viruses remained negative in both the WTR and the VR of the DIVA real-time PCR. For inclusivity, a number of classical (n = 6), recombinant (n = 12), and vaccine LSDV strains (n = 4) were tested. All the strains/isolates were correctly identified by the new DIVA (Table 2).

Subsequently, the interaction with the other non-LSDV capripox viruses (18 virulent + 5 vaccine strains) was evaluated by testing virus stocks of sheeppox virus (SPPV) and goatpox virus (GTPV) wild-type and vaccine strains. In the initial analyses, some high titer SPPV and GTPV stocks displayed a weak (borderline) positive signal in the WTR (Table 3). The extent of this cross-reaction was examined by testing a dilution series of the cross-reacting SPV’s and GPV’s. Cross-reaction in the WTR was seen up to a Ct value of 31 in the D5R in some strains.

The absence of a cross-reaction with SPPV strains at moderate Ct values was confirmed by testing 36 samples (blood, skin, and M. masseter: 12 each) from SPPV-infected sheep with the new DIVA PCR. The three SPPV strains used for infection of those sheep had been shown to cause a weak cross ratio when the pure viral stocks were tested. Although all the samples were positive on the pan capripox diagnostic PCR (Ct range 27.3 to 45; average 32.5 with a standard deviation of 4) and considered positive for SPPV, they remained all negative on the DIVA real-time PCR.

### 3.4. Impact of Joint Presence of Wild-Type and Vaccine LSDV Strains

Since infections with wild-type strains can occur in recently vaccinated animals, the impact of the simultaneous presence of wild-type (classic or recombinant) and vaccine strains on the performance of the DIVA PCR was tested.

This was achieved by diluting DNA samples of LSDV Bulgaria (classical wild-type LSDV), Neethling vaccine strain, and the recombinant isolate of Vietnam to a similar Ct value (Ct 28.51 standard deviation: 0.16). Mixes of wild-type (classical or recombinants) and vaccine DNA samples were made whereby one was kept constant while the other was decreased by 10-fold. There were three replicates of each dilution series that were tested. The impact on the Ct-value on the most abundant strain was almost nil in all the mixes tested. For the minority strain, however, the LOD increased significantly compared to when this is the only strain present. Wild-type strains (classical and recombinants wild-type) could be detected in an up to a 10-fold abundance of the vaccine strain. The Neethling vaccine could still be correctly detected in a 100-fold abundance of wild-type strains (classical and recombinant) but not always in a 1000-fold abundance (Table S3).

### 3.5. Intra- and Inter-Run Variability

For the intra- and inter-run variability, two dilutions (medium positive: Ct around 28; weak positive: Ct between 35 to 38) of a classical wild-type, recombinant wild-type and Neethling vaccine strain were prepared. A total of four replicates of each dilution were tested on five different days. The intra-run variability was very low for all three LSDV strains, although it was slightly higher for the weak positive dilution, even though the variability between repeats remained within 1 Ct. Similarly, the inter-run variability was very low for all three LSDV strains with a %CV below 1.5%. The data are summarized in Table 4.

## 4. Discussion

The availability of reliable diagnostic tools is important, particularly in disease outbreak situations. They are used for the early detection of the pathogens in the population but can also be important when vaccination is used to control the disease. The ability to discriminate between vaccinated and infected animals (DIVA principle) is important to avoid trade restrictions and make the control sustainable over a longer period of time. The emergence of LSDV recombinants since 2017 has impaired the control of LSDV. The current available DIVA diagnostic tools cannot distinguish been the recombinant wild-type LSDV strains and the Neethling-based vaccine strains which are used by most live attenuated commercial vaccines. Although these vaccines have been proven to be efficient [26,27], a Neethling-like response has been reported in the past in a limited number of animals whereby nodules are seen and where the vaccine strain can be detected in these nodules as well as in the blood or secretions, such as milk [27,34,35]. This reinforces the need for diagnostic DIVA tools when these vaccines are used. The reason that the previously developed DIVA tools have problems differentiating the recombinant LSDV strains from the Neethling vaccine strains is caused by the fact that one of the parental strains of the recombinants is a Neethling-like vaccine strain. One potential approach to solve this problem is put forward by Wolff et al. [36]. A duplex real -time PCR, specifically targeting the Neethling vaccine and “classic” wild-type strains, is combined with a general capripox PCR. A positive signal in the latter and a negative result in this duplex could be indicative of the presence of a recombinant LSDV strain. Although this is an interesting and viable option, additional testing is needed to confirm the presence of a potential recombinant wild-type LSDV strain.

We, therefore, attempted to develop a duplex real-time PCR that was capable of detecting the Neethling-like vaccine strains on the one hand and the classical (cluster 1.2) and recombinant wild-type LSDV strains that are currently circulating in Asia (cluster 2.5) on the other hand. This addresses the most urgent diagnostic question in the ongoing LSDV epidemic in Asia, driven by the recombinant strain. From a control/eradication or preventive perspective, it is important to know whether you are dealing with a vaccine or a virulent strain. When the newly developed DIVA PCR indicates that a wild-type strain is circulating, additional characterization is still needed to determine if it is a classical or a recombinant wild-type strain The newly developed assay is designed to detect, aside from the classical wild-type strains (cluster 1.2), the recombinant lineage which has become dominant since 2020 and is currently circulating in Asia (cluster 2.5) and Russia [21]. Based upon the in silico analysis of the primers/probes, the new DIVA real-time PCR should also correctly detect some recombinants which have been identified prior to 2020, including Saratov 2017/2019 (cluster 2.1) and Udmurtiya 2019 (cluster 2.2). The strains belonging to recombinant cluster 2.3 (represented by the Konstanay isolate) and 2.4 (Tyumen isolate) have important mismatches in the primer and/or probe regions, making it highly likely that they will not be detected by the new DIVA. However, this is based on the in silico evaluation only and needs further verification. As the new DIVA PCR is to be used in conjunction with a first line pan capripox PCR, this lack of detection can still provide some information similar to the approach of Wolff et al. [36]. The detection of the South-African isolates, identified by Van Schalkwyk from 1954 (Haden [37]) and the 1990′s [38], remain challenging. These field isolates are part of cluster 1.1 together with the Neethling-based vaccines and are typed as such by the new DIVA. However, these isolates seem to be confined to the region where they were identified, similar to the recombinants from cluster 2.1 to 2.4, as no spreading has been observed in recent years. Therefore, these limitations of the new DIVA PCR do not impede its use in Asia to control the current LSDV epidemic. However, new genomic sequences and analyses are added to Genbank/PubMed at a very regular basis from multiple geographically regions. For the moment, it is not possible to predict how this will further evolve and if all recombinants have been characterized so far or new ones will still arise. Therefore, caution and a continued vigilance is warranted whereby DIVA tools are constantly evaluated and updated if needed. Aside the new recombinant strains, the in silico analysis of the newly developed DIVA PCR shows that the new strains from India represented by the Genbank sequences OK422493 and ON400507 are correctly classified as wild-type, although these Indian sequences cluster more closely around historical South African NI2490 strains and KSGP-like strains from Kenya [39,40].

The new DIVA PCR was designed as a confirmatory typing assay, to be used in combination with a first line detection test. Nevertheless, good performance of the assay for parameters such as LOD, ASe, ASp, DSe, DSp, as well as inter- and intra-run variability were obtained during the validation. The high sensitivity is evidenced by the comparison of the new DIVA with diagnostic pan capripox PCR described by Haegeman et al. [33] using different organs/tissue matrices over a large range of Ct values. The fact that very similar Ct values are obtained for WTR as well as VR compared to D5R supports the high sensitivity reported for Ase. With the exception of a limited cross-reaction with some SPPV and GTPV strains (when present at viral loads only attainable in virus stocks), the specificity (analytical and diagnostic) of this new DIVA PCR is high. Although only isolates of Vietnam are used in the evaluation of this new PCR, the in silico analysis shows clearly that the targeted sequences are 100% conserved among the isolates from Vietnam, Thailand, Taiwan, and China. It can, therefore, with high confidence, be stated that the other isolates will be correctly identified as well. The cross-reaction with SPPV and GTPV is only observed in cases of high viral load present in the sample. As this assay is to be used for LSDV detection in cattle, this cross-reaction is not a problem as high genomic load of SPPV and GTPV in cattle is not encountered. Our results showed that samples from SPPV-infected sheep remained negative in the developed DIVA PCR further supports our hypothesis that this will not lead to problems in field conditions. Vaccination with heterologous SPPV- and GTPV-based vaccines could result in the presence of SPPV or GTPV sequences that could be detected as this has been observed for other viruses [41,42]. However, results from unpublished vaccination/infection trials under controlled and standardized conditions with commercial SPPV- (n = 4) and GTPV-based (n = 1) live attenuated vaccines show that no traces of the vaccine genome were found in the vaccinated animals. This supports further the notion that the observed cross-reaction does not hamper the use of this new DIVA PCR.

The developed DIVA real-time PCR method is furthermore able to correctly identify the simultaneous presence of wild-type (classic, recombinant) and Neethling-based vaccine strains as long as the wild-type strain concentration is at least 10% of that of the vaccine strain, or the vaccine strain is at least present at 1% of the wild-type strain. The difference in simultaneous detection between the wild-type and vaccine strains is most likely explained by the fact that the forward primer of the WTR is conserved among wild-type and vaccine strains. This can result in competition for the primers. A similar phenomenon is observed in the DIVA PCR published by Agianniotaki et al. [28]. The risk of not detecting a wild-type strain (classic or recombinant) due to a high abundance of a vaccine strain is theoretically possible when an LSDV infection occurs shortly after the vaccination. The presence of the vaccine strain in the blood has been demonstrated in a part of the vaccinated animals [27] but this is limited in time [up to the first 2 weeks after vaccination]. This can be longer in skin biopsies of animals with a Neethling-response as the small nodules/wound crust can persist longer. However the prevalence of such a Neethling response is low in general varying from 0.1 to 0.4%, and up to 1.5% [43,44,45] of the vaccinated animals.

In summary, we report the development of a new DIVA real-time PCR that is able to differentiate between classical and recombinant wild-type LSDV strains on the one hand, and Neethling-based vaccine strains on the other. The validation parameters for this newly developed test were promising, indicating that it can be an important diagnostic tool to help to combat the current LSDV epidemic in Asia.

## Figures and Tables

**Table 1:** Probe and primer sequences for the WTR and VR; +: an LNA nucleotide.

Wild-Type Reaction (WTR)	
WTR_Frw	5-GGAATCTGTGCAGAAATAAAGTACGA-3
WTR_Rev	5-CCGAAGGGAACGCACTG-3
WTR_Pr	56-FAM-+CTCATCAAATCCCTCTATTTTATG-BHQ1-3
Vaccine Reaction (VR)	
VR_Frw	5-GGATTTATTTATATTGTGGGTGGAATT-3
VR_Rev	5-TTTTTGTATGTCGTAATGGGTTC-3
VR_Pr	5-HEX-CTCTCGGAATAGGCTATGAAGG-BHQ1-3

**Table 2:** Real-time PCR results for a range of classical wild-type, recombinant type, and Neethling vaccine strains (WTR: wild-type reaction; VR: Neethling vaccine type reaction). The results are expressed as Ct values.

Classical Wild-Type LSDV	WTR	VR
LSDV Israel	22.30	Neg
LSDV Cyprus	25.62	Neg
LSDV Kazakhstan	22.58	Neg
LSDV2	26.69	Neg
LSDV Greece, Evros	21.37	Neg
LSDV Bulgaria	21.91	Neg
LSDV KSGP 0240 (Kenyavac, Jovac,)	23.35	Neg
New Recombinants		
Vietnam isolate Zol58	15.86	Neg
Vietnam isolate zol79	28.89	Neg
Vietnam isolate zol70	17.04	Neg
Vietnam isolate zol68	15.97	Neg
Vietnam isolate zol45	22.86	Neg
Vietnam isolate zol81	19.02	Neg
Vietnam isolate zol50	15.76	Neg
Vietnam isolate zol48	14.98	Neg
Vietnam isolate zol42	18.14	Neg
Vietnam isolate zol62	20.86	Neg
Vietnam isolate zol43	15.76	Neg
Vietnam isolate zol15	20.80	Neg
Neethling vaccine strains		
OBP	Neg	19.49
LSDV Neethling strain	Neg	20.54
Lumpyvax MSD	Neg	22.30
Herbivac Deltamune	Neg	34.27

**Table 3:** Real-time PCR results of sheep- and goatpox strains. Bold: the undiluted sample.

	DIVA		D5R		DIVA		D5R
	WTR	VR			WTR	VCR	
**SPPV B1/10**	38.24	Neg	19.20	**SPPV Romanian**	Neg	Neg	32.41
SPPV B1/10 dil 10-1	Neg	Neg	23.01	**SPPV RM65 Arbic—Phibro**	Neg	Neg	22.16
**SPPV FSI**	39.86	Neg	17.55	**SPPV BK Poxdoll—Dolvet**	Neg	Neg	24.99
SPPV FSI dil 10-3	Neg	Neg	27.87				
**SPPV Pakistan**	Neg	Neg	23.07	**GTPV Gorgon pur**	36.76	Neg	21.13
**SPPV Kenyan**	41.10	Neg	23.30	GTPV Gorgon 10-1	Neg	Neg	23.97
SPPV Kenyan dil 10-2	Neg	Neg	31.12	**SGPV Kano**	Neg	Neg	24.32
**SPPV AEPT**	43.17	Neg	15.59	**SGPV Yemen**	Neg	Neg	21.94
SPPV AEPT dil 10-3	Neg	Neg	26.87	**SGPV Kedang**	Neg	Neg	22.60
**SPPV Turkish**	44.12	Neg	22.93	**SGPV Sudan**	Neg	Neg	24.24
SPPV Turkish dil 10-2	Neg	Neg	30.75	**GTPV Bangladesh**	41.67	Neg	26.61
**SPPV ABV Garib**	42.78	Neg	21.20	GTPV Bangladesh dil 10-1	Neg	Neg	30.81
SPPV ABV Garib dil 10-2	Neg	Neg	30.99	**GTPV Isiolo**	Neg	Neg	19.64
**SPPV 545**	45.00	Neg	21.68	**GTPV Indian**	37.51	Neg	20.15
SPPV 545 dil 10-2	Neg	Neg	28.79	GTPV Indian dl 10-3	Neg	Neg	30.38
**SPPV Nigeria**	39.07	Neg	22.56				
SPPV Nigeria dil 10-2	Neg	Neg	29.07				
**SPPV Saudia Arabia Vacine**	Neg	Neg	22.35				
**SPPV Arbel 2000**	Neg	Neg	25.04				

**Table 4:** The intra- and inter-run variability results of the DIVA real-time PCR. Ct values and %CV of five runs of four repeats of a low and high dilution of classical and recombinant wild-type strains (WTR) and vaccine strains (VR).

	DIVA PCR (a)					Standard Deviation						%CV					
Variability	Averaged Cp					Intra Run					Inter Run	Intra Run					Inter Run
	Run 1	Run 2	Run 3	Run 4	Run 5	Run 1	Run 2	Run 3	Run 4	Run 5		Run 1	Run 2	Run 3	Run 4	Run 5	
Classic virulent strain																	
Dilution 1	28.22	28.55	28.36	28.54	28.6	0.22	0.16	0.07	0.03	0.07	0.15	0.77	0.56	0.25	0.12	0.26	0.51
Dilution 2	39	37.82	38.56	38.02	37.91	0.96	0.36	0.37	0.39	0.33	0.45	2.45	0.94	0.97	1.03	0.87	1.18
Neethling vaccine strain																	
Dilution 1	28.13	28.34	28.27	28.23	28.21	0.12	0.22	0.22	0.26	0.25	0.07	0.43	0.76	0.76	0.92	0.87	0.24
Dilution 2	37.72	36.56	36.69	37.19	37.10	0.17	0.81	0.59	0.86	0.61	0.41	0.45	2.21	1.60	2.31	1.64	1.09
Recombinant strain																	
Dilution 1	28.66	28.47	28.51	28.69	28.58	0.08	0.09	0.05	0.05	0.07	0.08	0.27	0.31	0.18	0.19	0.25	0.29
Dilution 2	35.40	35.31	35.60	35.51	35.57	0.23	0.14	0.30	0.15	0.03	0.11	0.66	0.39	0.83	0.43	0.09	0.31

## Data Availability

The main data presented in this study are available within the study itself and other data may be made available through contact with the corresponding author.

## Supplementary Materials

The following supporting information can be downloaded at: https://www.mdpi.com/article/10.3390/v15040870/s1, Figure S1: Alignment capripox sequences in relation to the wild-type reaction (WTR) (primers/probe); Figure S2: Alignment capripox sequences in relation to the vaccine reaction (VR) (primers/probe); Figure S3: Linearity (4 replicates) of classical wild-type Bulgaria strain (A); Neethling-like vaccine strain (B) and a recombinant strain (C); Figure S4: Comparison of the differences ion Ct values between pan capripox real-time PCR and the new DIVA for LSDV wild-type positive samples; Figure S5: Comparison of the different ion Ct values between pan capripox real-time PCR and the new DIVA for Neethling-like vaccine-type-positive samples; Table S1: Comparison of real-time PCR results (Ct values) between pan capripox real-time PCR, the new DIVA and the DIVA published by Agianniotaki et al. [28] for LSDV wild-type blood/organ-positive samples; Table S2: Comparison of real-time PCR results (Ct values) between pan capripox real-time PCR, the new DIVA, and the DIVA published by Agianniotaki et al. [28] Neethling-like vaccine-type positive organ samples; Table S3: Real-time PCR results (3 replicates) of samples containing different combinations of Neethling-like vaccine strain/classic or recombinant LSDV in different ratios.
