# Supplementary figures and images for: Development and Validation of a New DIVA Real-Time PCR Allowing to Differentiate Wild-Type Lumpy Skin Disease Virus Strains, Including the Asian Recombinant Strains, from Neethling-Based Vaccine Strains
Source: Viruses. 2023 Mar 28;15(4):870. doi: 10.3390/v15040870 (PMC10146157; doi:10.3390/v15040870)

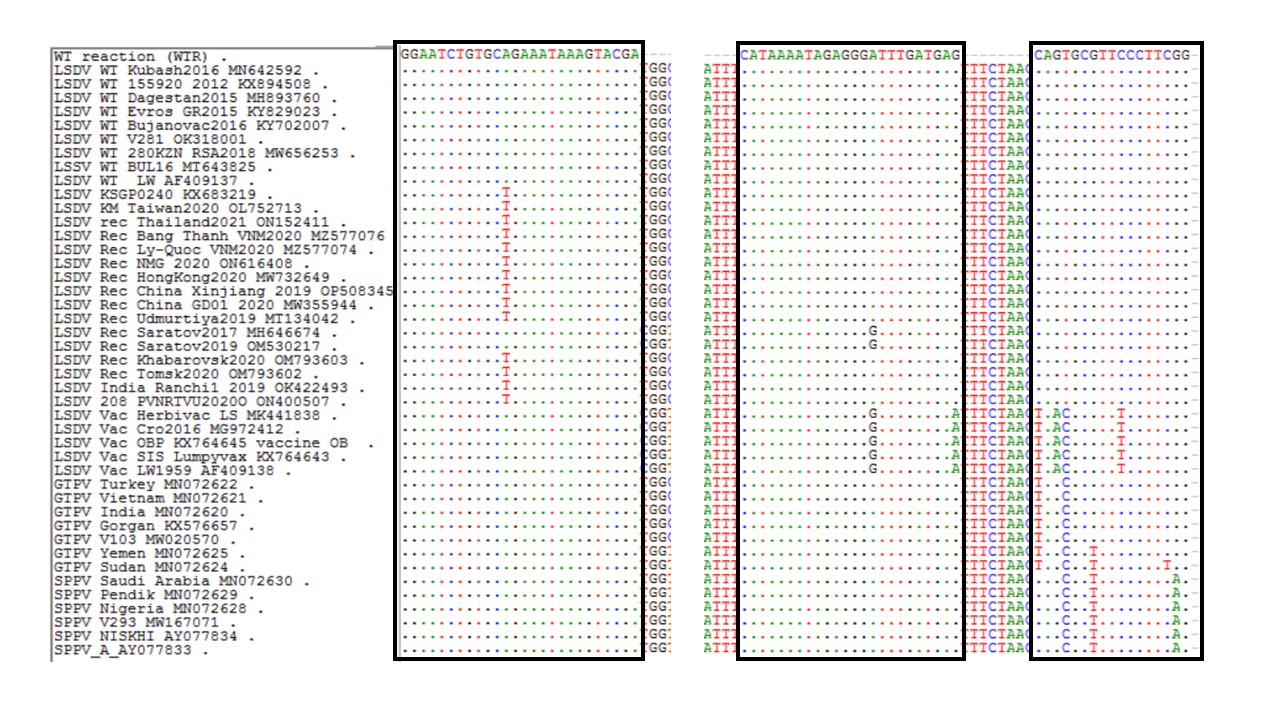

Supplement: Supplementary file 1 [file viruses-15-00870-s001.zip › Fig S1.jpg]

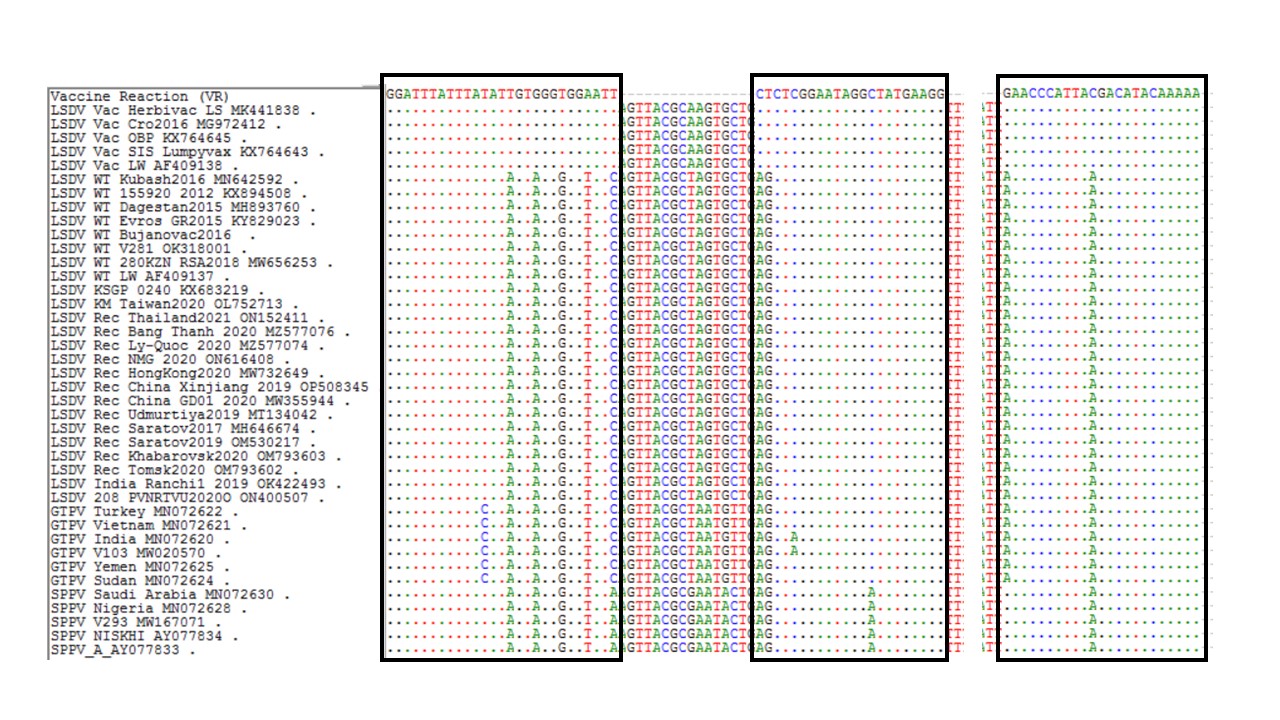

Supplement: Supplementary file 1 [file viruses-15-00870-s001.zip › Fig S2.jpg]

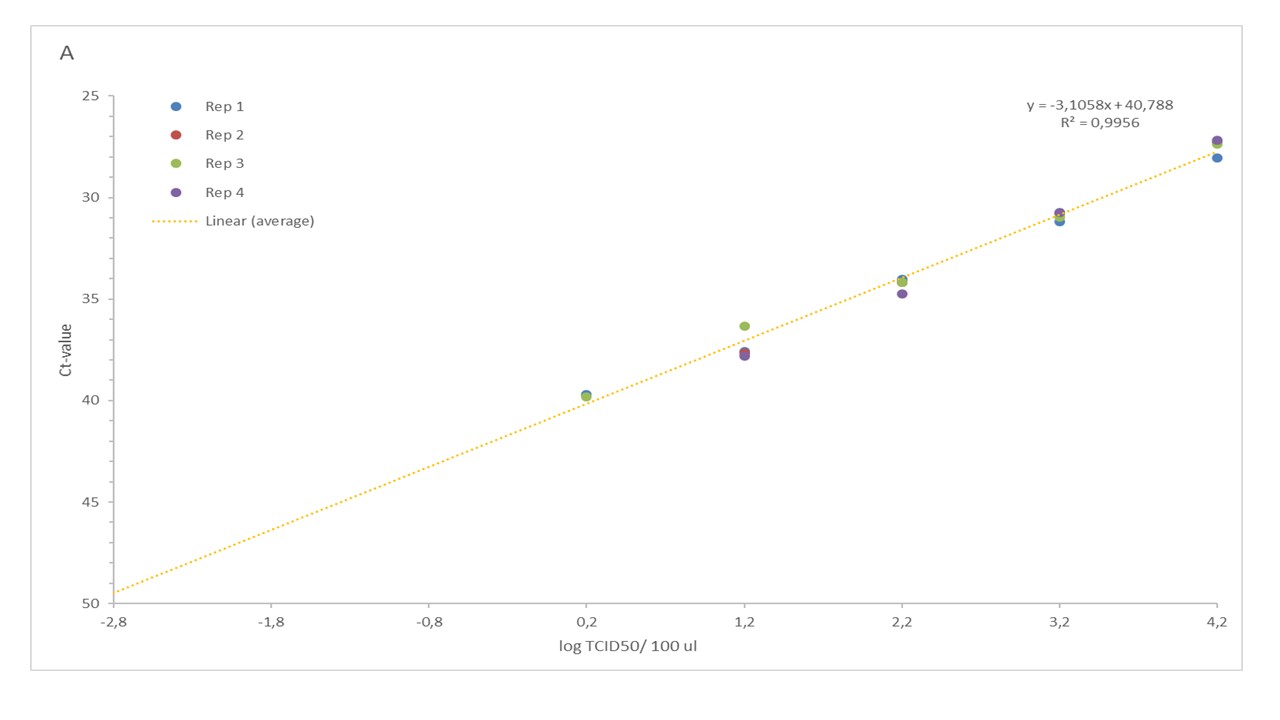

Supplement: Supplementary file 1 [file viruses-15-00870-s001.zip › Fig S3a.JPG]

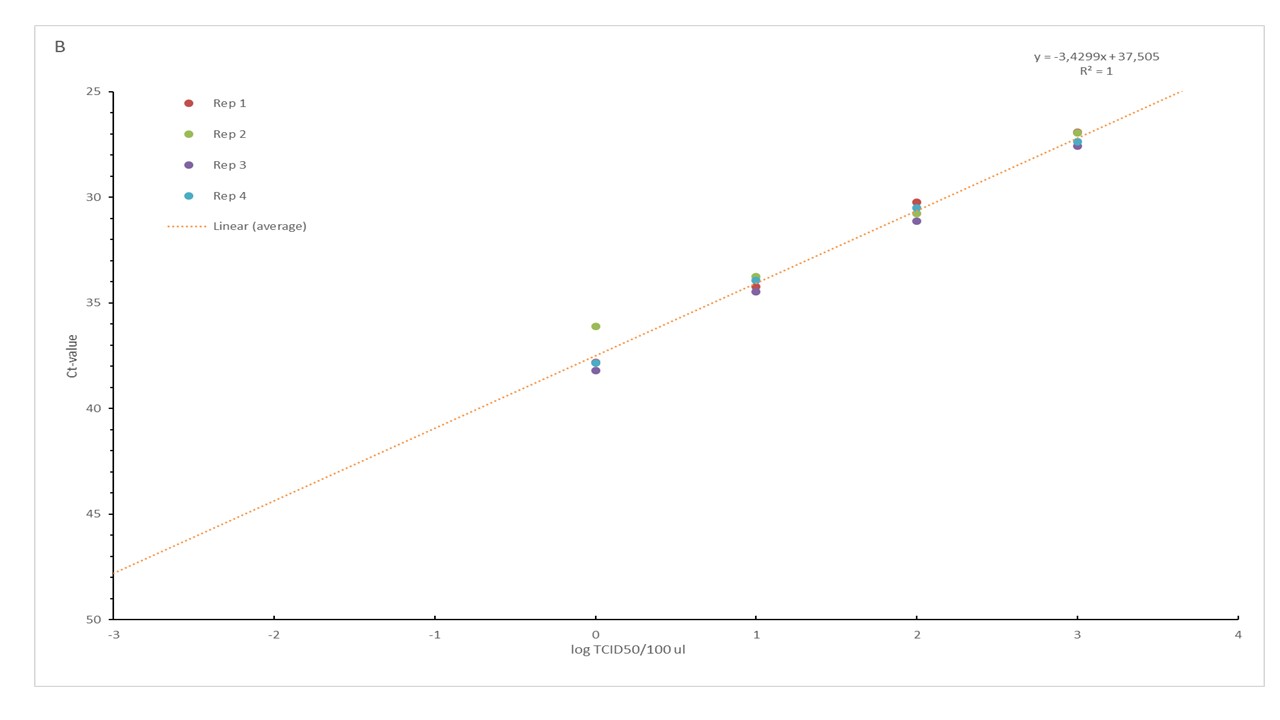

Supplement: Supplementary file 1 [file viruses-15-00870-s001.zip › Fig S3b.JPG]

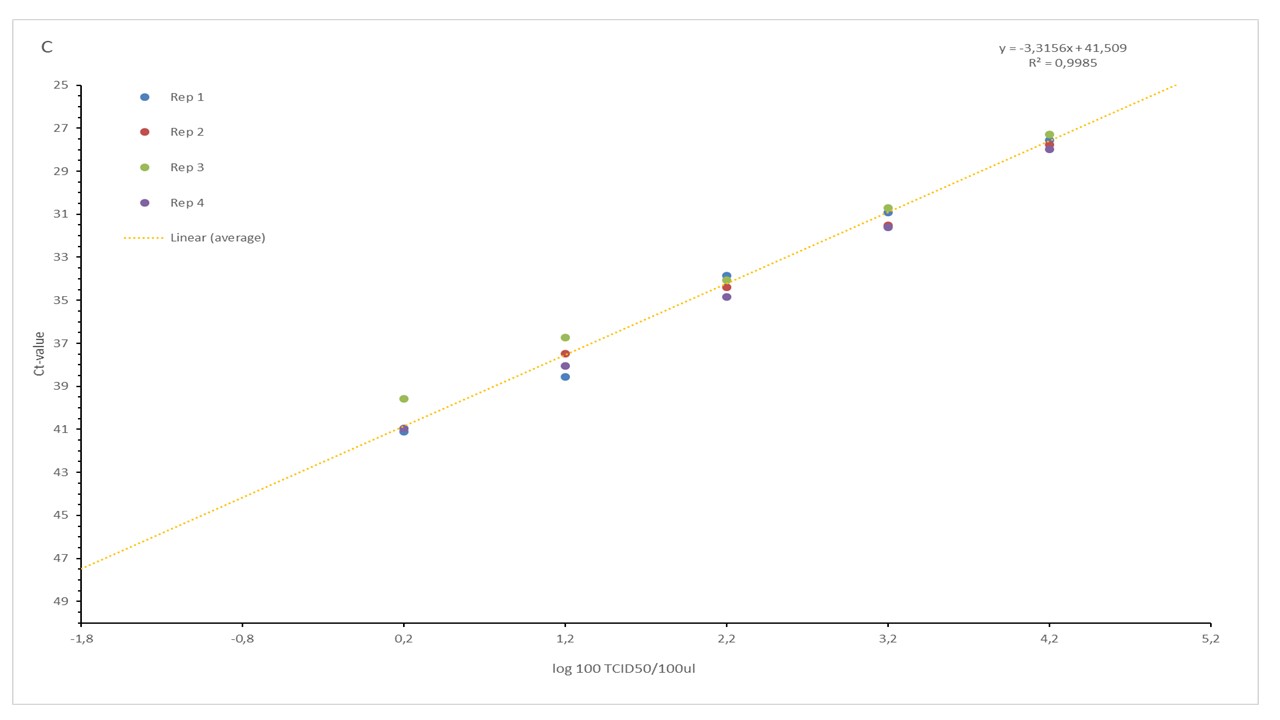

Supplement: Supplementary file 1 [file viruses-15-00870-s001.zip › Fig S3c.JPG]

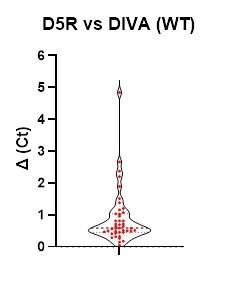

Supplement: Supplementary file 1 [file viruses-15-00870-s001.zip › Fig S4_300.jpg]

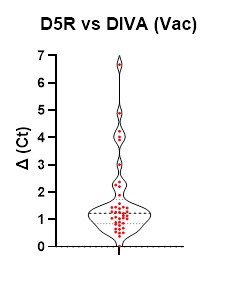

Supplement: Supplementary file 1 [file viruses-15-00870-s001.zip › Fig S5.jpg]

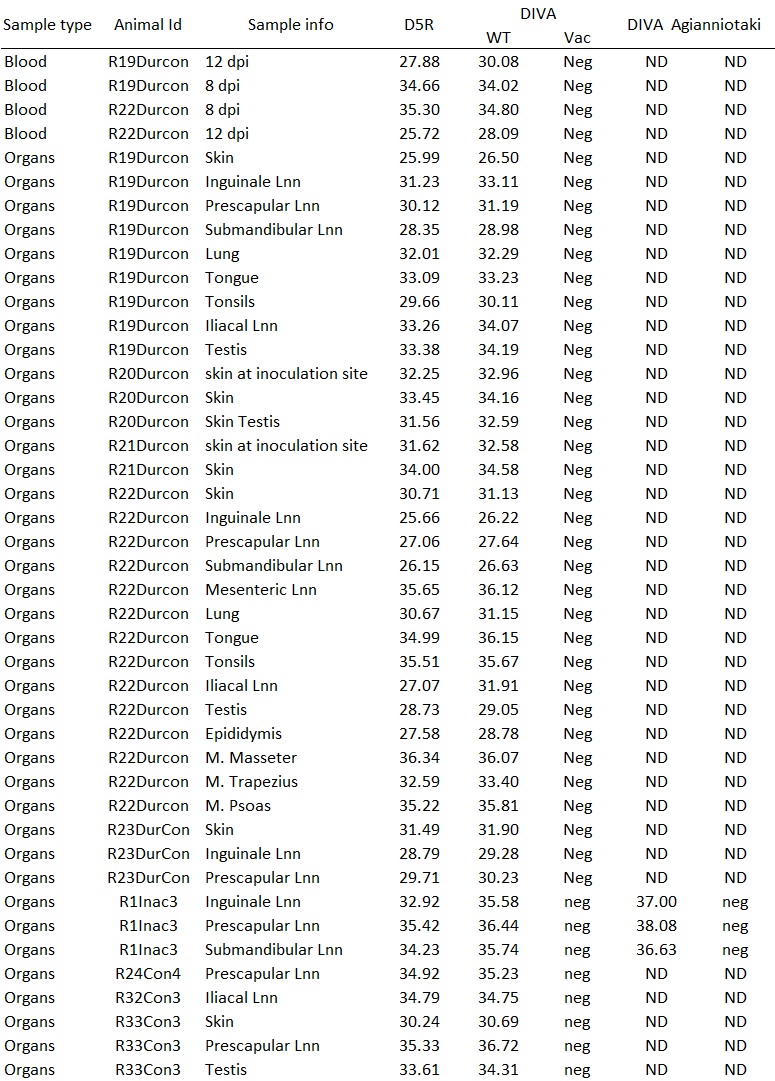

Supplement: Supplementary file 1 [file viruses-15-00870-s001.zip › Table S1.jpg]

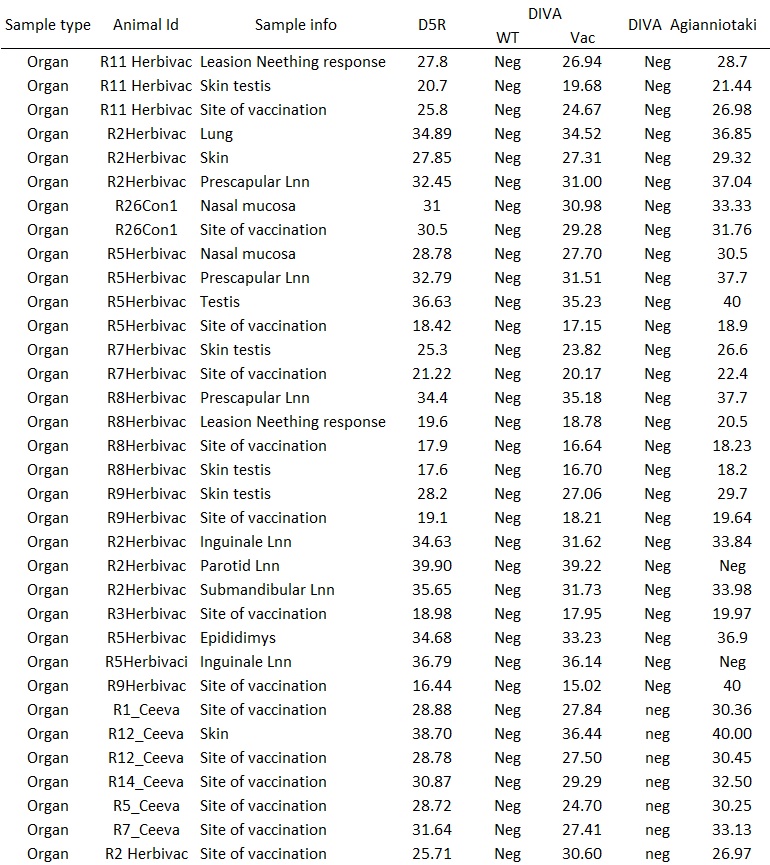

Supplement: Supplementary file 1 [file viruses-15-00870-s001.zip › Table S2.jpg]

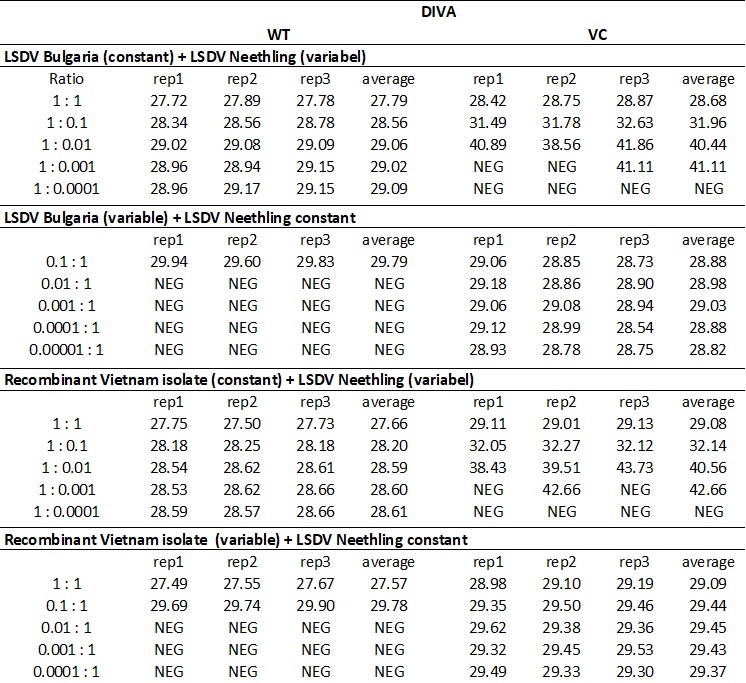

Supplement: Supplementary file 1 [file viruses-15-00870-s001.zip › Table S3.jpg]
